# Azithromycin-chloroquine and the intermittent preventive treatment of malaria in pregnancy

**DOI:** 10.1186/1475-2875-7-255

**Published:** 2008-12-16

**Authors:** R Matthew Chico, Rudiger Pittrof, Brian Greenwood, Daniel Chandramohan

**Affiliations:** 1 Department of Infectious and Tropical Disease, London School of Hygiene and Tropical Medicine, Keppel Street, London, WC1E 7HT, UK; 2 Enfield Town Clinic, Wenlock House, 33 Eaton Road, Enfield, EN1 1NJ, UK

## Abstract

In the high malaria-transmission settings of sub-Saharan Africa, malaria in pregnancy is an important cause of maternal, perinatal and neonatal morbidity. Intermittent preventive treatment of malaria in pregnancy (IPTp) with sulphadoxine-pyrimethamine (SP) reduces the incidence of low birth-weight, pre-term delivery, intrauterine growth-retardation and maternal anaemia. However, the public health benefits of IPTp are declining due to SP resistance. The combination of azithromycin and chloroquine is a potential alternative to SP for IPTp. This review summarizes key *in vitro *and *in vivo *evidence of azithromycin and chloroquine activity against *Plasmodium falciparum *and *Plasmodium vivax*, as well as the anticipated secondary benefits that may result from their combined use in IPTp, including the cure and prevention of many sexually transmitted diseases. Drug costs and the necessity for external financing are discussed along with a range of issues related to drug resistance and surveillance. Several scientific and programmatic questions of interest to policymakers and programme managers are also presented that would need to be addressed before azithromycin-chloroquine could be adopted for use in IPTp.

## Background

Each year, 30 million pregnancies are at risk of malaria infection in sub-Saharan Africa, representing a major public health problem. Malaria in pregnancy (MIP) is associated with low birth-weight (LBW) [1-3], pre-term delivery [4] intrauterine growth-retardation [4,5], and maternal anaemia [6]. LBW is a strong predictor of infant mortality in sub-Saharan Africa; death within the first year of life is three-times higher for LBW newborns compared to neonates of normal birth-weight [7]. Malaria is one of the few contributors to LBW that can be improved by specific interventions [8]. Thus, to reduce the effects of MIP in endemic areas, the World Health Organization (WHO) recommends use of Intermittent Preventive Treatment of Malaria in Pregnancy (IPTp) with sulphadoxine-pyrimethamine (SP).

IPTp has two primary objectives: (1) to clear asymptomatic peripheral and placental parasitaemia and (2) to provide intermittent chemoprophylaxis against malaria infection during pregnancy. The WHO recommends administration of two or three courses of SP, sulphadoxine (500 mg) and pyrimethamine (25 mg), after foetal quickening with each course given no less than one month apart, and all prior to the last month of pregnancy [9]. Anti-malarial chemoprophylaxis among paucigravidae increases birth-weight by an average of 127 g (95% CI 88.64 to 164.75 g) and reduces, by nearly half, the incidence of LBW (RR = 0.57, 95% CI 0.46 to 0.72) [10]. It has been estimated that universal coverage with IPTp would reduce all-cause neonatal mortality by 32% (95% CI -1 to 54%) [11].

Standard IPTp dosing does not provide the same degree of protective efficacy for pregnant women who are HIV-positive. This can be overcome, however, by administering more frequent courses of SP throughout pregnancy. A study in Malawi compared monthly SP treatment versus two courses during the antenatal period among HIV-positive and HIV-negative women. An estimated 7.8% of HIV-positive pregnant women had placental malaria after receiving monthly SP as compared to 21.5% of HIV-positive women who received two doses of SP (RR, 0.36 [95% CI, 0.17–0.79]). Among HIV-negative women, 2.3% who received monthly SP had placental parasitaemia in contrast to 6.3% of HIV-negative women who received two-dose SP (RR, 0.37 [95% CI, 0.11–1.19]) [8].

The benefits of IPTp are threatened by increasing resistance of *Plasmodium falciparum *to SP. In many countries, artemisinin combination therapy (ACT) has replaced SP for case management, according to WHO guidelines, because SP now demonstrates inadequate therapeutic efficacy in children [12]. Therapeutic efficacy of SP in children with clinical cases of *P. falciparum *malaria does not, however, predict efficacy of IPTp. Correlation analysis between paediatric treatment and IPTp suggests that SP may still offer some protection against MIP in geographic areas where day 14 post-treatment failure rates for SP in children are as high as 40% [13]. This protection, however, is not uniform across populations; primigravidae are particularly vulnerable to the effects of MIP and are protected the least by SP where sensitivity is on the decline. In Ghana, where parasite sensitivity to SP remains higher than in east and southern Africa, uncorrected parasitological failure rates by day 28 post-treatment were 36.4% (32 of 88) in children, 27.1% (29 of 107) in primigravidae, 6.1% (3 of 49) in secundigravidae, and 3.8% (2 of 52) in multigravidae [14]. Thus, SP is already compromised and an urgent need exists to identify alternative compounds for use in IPTp, even though SP still offers some protection up to an unknown threshold of parasite resistance.

An ideal anti-malarial drug or drug combination for IPTp should be safe, well tolerated, efficacious in the clearance of malaria parasites, provide a long period of chemoprotection and, preferably, not be deployed as the first-line treatment for clinical malaria. If a drug or drug combination used in IPTp is not simultaneously used for clinical malaria case management, then IPTp may actually lower the selection pressure on the first-line drug by decreasing symptomatic cases that require treatment. This is particularly important as countries increasingly treat pregnant women with uncomplicated clinical malaria in second- and third-trimesters with ACTs.

Several published reviews of drugs for IPTp have included azithromycin and chloroquine, although only as monotherapies [15-18]. This review summarizes *in vitro *and *in vivo *evidence for the therapeutic efficacy of azithromycin and chloroquine when used alone or together and discusses the additional benefits that the combination could have on many sexually transmitted diseases and, possibly, pneumococcal infection during pregnancy. Drug costs are presented along with issues related to drug resistance and surveillance. Several scientific and programmatic areas are outlined, as well, that would need to be addressed for policymakers and programme managers before azithromycin-chloroquine could be adopted for use in IPTp.

### Azithromycin monotherapy for treatment and prevention of malaria

#### Pharmacokinetics

Azithromycin is a slow-acting anti-malarial macrolide [19], an analogue of erythromycin with a nitrogen atom inserted into the macrolide nucleus. As a result, there is enhanced penetration of drug into macrophages, fibroblasts and polymorphonuclear neutrophils, permitting greater accumulation within acidified vacuoles and extending the 1.5-hour half-life of erythromycin to 68 hours for azithromycin [20]. Stable at gastric pH, azithromycin has an absolute bioavailability of 37% following oral administration [21]. It accumulates in hepatic, renal, pulmonary and splenic tissue [22], and gradually leaches into the bloodstream over a period of one week [23]. Mild renal dysfunction and mild-to-moderate hepatic dysfunction do not affect excretion significantly.

Among pregnant women, serum concentrations peak within six hours of oral administration and are sustained for 24 hours. As the drug disperses, peak concentrations are maintained three-times longer in the placenta, myometrial and adipose tissues [24]; only 2.6% of a maternal dose, however, perfuses the placenta [25]. Azithromycin is excreted in human milk with no adverse events observed as a consequence [26].

Azithromycin targets the 70 S ribosomal subunit of the apical complex of susceptible micro-organisms including *P. falciparum *and *P. vivax *[23]. Once attached to the apicoplasts of the parasite, azithromycin hinders polypeptide development, triggering premature detachment and movement down the peptide exit tunnel. The potency of azithromycin, as a translation inhibitor, is greatest against the progeny of parasites that inherit a non-functioning apicoplast following drug exposure, thus creating a delayed-death effect [27-29]. Laboratory-generated *P. falciparum *that are resistant to azithromycin appear to accumulate mutations after *in vitro *passage in the structural proteins of the ribosome [23]. It is unknown whether mutations induced by azithromycin are capable of undermining the inhibitory action of other drugs that also target the apicoplast.

#### Safety and tolerability

Doses of azithromycin between 500 mg and 2,000 mg have been used in all trimesters of human pregnancy for the treatment of upper and lower respiratory tract infections, skin diseases, *Chlamydia trachomatis*, mycoplasma and group B streptococci infections among women allergic to other antibiotics.

Meta-analysis of eight randomized clinical trials among pregnant women with *C. trachomatis *infection found that azithromycin was associated with fewer gastrointestinal adverse events than erythromycin (OR = 0.11, 95% CI 0.07–0.18) and fewer total adverse events (OR = 0.11, 95% CI 0.07–0.18) [30]. A placebo-controlled trial, however, suggests that azithromycin may be poorly tolerated by HIV-positive patients. Gastrointestinal effects were reported by 78.9% of azithromycin recipients (67 of 85) and 27.5% of participants given placebo (24 of 89) [31]. Although an unusual side-effect, case reports indicate that HIV-positive patients may experience temporary ototoxicity with azithromycin use [32].

Adults treated with a 1,000 mg oral dose of azithromycin report moderate side-effects including diarrhoea or loose stools (7%), nausea (5%), vomiting (2%), and vaginitis (2%); up to 1% of adults experience dizziness, headache, vertigo, and somnolence [33]. There is no evidence of teratogenicity in animal models, even at four-times the human treatment dose [34-36].

#### Efficacy

The *in vitro *anti-malarial activity of azithromycin increases 200-fold against *P. falciparum *isolates when incubated between 24 and 48 hours, while its 50% inhibitory concentration values drop as low as 35 nanomolar [37]. At 48-hours, azithromycin is 10-fold more active than erythromycin against chloroquine-resistant *P. falciparum*; the two compounds are equipotent, however, when chloroquine-sensitive parasites are exposed to the same drug concentration [38,39].

Daily regimens of 250 mg with a loading dose of 500 or 750 mg have shown an impressive chemoprophylactic effect against *P. vivax*. Azithromycin had a 99% protective effect (95% CI, 93% to 100%) among semi-immune subjects in Indonesia over a 20-week period [40]. A similar protective efficacy, 98% (95% CI, 88% to 100%), was seen in Thailand [41] in a semi-immune population. By comparison, the chemoprophylactic effect of azithromycin against *P. falciparum *has shown promise, but has been less impressive (Table 1).

**Table 1:** Chemoprophylactic effect of azithromycin monotherapy against *Plasmodium falciparum *in semi-immune non-pregnant adults.

**Country**	**Treatment regimen**	**Sample size**	**Duration of follow-up**	**Chemoprophylactic effect (95% CI)**
Kenya [40]	250 mg daily	59	10 weeks	83% (68–91)

Kenya [40]	1,000 mg weekly	58	10 weeks	64% (47–77)

Indonesia [36]	750 mg loading dose and 250 mg daily	148	20 weeks	72% (50–84)

Thailand [37]	750 mg loading dose and 250 mg daily	179	20 weeks	71% (-14–94)

The first published *P. falciparum *human challenge study with azithromycin involved four non-immune volunteers who received a loading dose of 500 mg and 250 mg daily for three days. Subjects were then inoculated with five *Anopheles stephensi *mosquitoes that had an average of 3.2 salivary-gland *P. falciparum *sporozoites each, after which they continued to receive 250 mg of azithromycin daily for four more days. With unquantifiable plasma concentrations of azithromycin, presumably due to poor absorption, one of four subjects developed parasitaemia in the 14-day post-challenge period [42].

A subsequent trial suggested that a regimen of longer duration might be required against *P. falciparum*. Ten non-immune subjects were given a loading dose of 500 mg followed by 250 mg daily for 2 weeks prior to parasite exposure. After inoculation, 250 mg was administered daily for one additional week, producing a protective effect of 40% (95% CI, 12% to 74%). A concurrent human challenge study with 10 non-immune subjects was conducted using the same regimen, except that 250 mg was given daily for two weeks post-exposure, rather than for just one, producing a 100% protective effect [43]. This high level of protection has not been replicated in the field, however, where multiple infections may be expected.

Among semi-immune populations, an equivalent or higher loading dose with the same daily regimen resulted in protective efficacies of 83% (95% CI, 69% to 91%) in Kenya [44], 71% (95% CI, -14% to 94%) in Thailand [41], and 72% (95% CI, 50% to 84%) in Indonesia [40]. There were two sub-populations in the Indonesian study which may have had slightly different degrees of acquired immunity: the chemoprophylactic effect among soldiers living for six months in the study area was 62.9% (95% CI, 29.5 to 80.4) while chemoprophylactic protection among civilians residing in the study area for less-than 18 months was 88.4 (95% CI, 56.6 to 97.4). The Kenyan trial also evaluated weekly dosing with 1,000 mg of azithromycin, in contrast to a daily regimen of 250 mg, producing just 64% protective efficacy (95% CI, 47% to 77%) [44]. Despite relatively poor *in vivo *protection against *P. falciparum *in field settings, the favourable safety profile of azithromycin in pregnant women and young children prompted further investigation into molecules that might be co-administered with azithromycin to improve its protective effect.

### Chloroquine as monotherapy for the treatment and prevention of malaria

#### Pharmacokinetics

Chloroquine has been the first-line treatment of malaria in much of the world for most of the past 60 years. Absolute bioavailability is 70 to 75% while peak plasma concentrations are reached within two hours of oral administration. A single therapeutic dose against a chloroquine-resistant strain will persist six to 10 days in the blood, while its overall half-life is between one and two months [45,46]. Chloroquine accumulates extensively in the liver, connective tissue and pigmented tissue such as skin and retina, enabling enormous total volume distribution. Greatest concentrations are found in erythrocytes, granulocytes and platelets, and 55% is protein-bound in plasma.

Chloroquine is active against erythrocytic life stages of *Plasmodium *species when haemoglobin is being actively digested. Haem is a toxic bi-product of haemoglobin ingestion and must be eliminated through dimerization. Under normal circumstances, parasites bio-crystallize haem to form haemozoin, the iron-containing pigment that accumulates as non-toxic cytoplasmic granules. Chloroquine prevents this process by concentrating at nanomolar levels outside parasites (10^-9^) and one million times higher (10^-3^) in parasite food vacuoles of infected erythrocytes [47]. Inside parasite vacuoles, chloroquine binds to haem, preventing its expulsion. Thus, the more haemoglobin ingested by parasites, the more toxic their food vacuoles become, rapidly causing cell death. Resistance to chloroquine is associated with polymorphisms in the *P. falciparum *food vacuole transporter protein (*pfcrt*) located on chromosome 7 [48]. All *pfcrt *alleles from chloroquine-resistant strains, regardless of geographic origin, encode a conserved K76T amino acid substitution. The effect of *pfcrt *on chloroquine pharmacokinetics remains disputed. Some researchers have theorized that *pfcrt *enables protonated chloroquine to escape the food vacuole while others argue *pfcrt *binds directly to chloroquine, thereby inhibiting its ability to alter food vacuole pH [49].

#### Safety and tolerability

Chloroquine is safe and generally well tolerated in treatment doses. Due to its rapid absorption, chloroquine has a narrow therapeutic index, increasing the potential for toxic overdose. Hypotension and cardiac failure can be prompted by a single oral dose of 3500 mg [50]. Despite toxicity at high doses, the most commonly reported side-effect in African populations is pruritus which peaks 24 hours after intake [51]. Chloroquine has been used safely in all trimesters of human pregnancy for decades as both a treatment and chemoprophylactic drug, crossing the placenta without teratogenic effect [52].

Prior to establishment of IPTp in sub-Saharan Africa, chloroquine was commonly given to pregnant women during antenatal visits in sachets containing four weekly doses of 300 mg for self-administration. Compliance with chloroquine remained low for many reasons including pruritus and its bitter taste which some women associate with medications that induce abortion [53,54].

#### Efficacy

Although still a first-line treatment for *P. vivax*, chloroquine is no longer recommended for treatment of *P. falciparum *due to high levels of resistance. In combination with another anti-malarial drug, however, chloroquine might, once again, have a role in malaria control. Malawi changed its first-line drug to SP in 1993 when chloroquine *in vivo *failure rates were as high as 58% [55]. Five years later, chloroquine inhibited *in vitro *blood schizont development in 96.5% (28 of 29) of isolates from Malawi [56], indicating that *pfcrt *was no longer under selection pressure. In 2001, field sampling failed to find parasites carrying the *pfcrt *mutation associated with resistance [57] and a clinical trial using chloroquine monotherapy was 100% efficacious (63 of 63) among asymptomatic semi-immune adults who received 600 mg on day 0 and day 1, and 300 mg on day 2 [58]. Most recently, a study in 2005 showed chloroquine to clear parasite infection in 98.8% (79 of 80) of Malawi children with uncomplicated *P. falciparum *malaria [59].

The re-emergence of high *in vitro *sensitivity to chloroquine in Malawi – within just five years – suggests the *pfcrt *resistance mutation involves considerable fitness cost to *P. falciparum *[60-62]. This micro-evolutionary reversal is all the more remarkable because it occurred despite the continued availability of chloroquine in the formal and informal private sector. It is likely that *P. falciparum *sensitivity will return elsewhere in the region, if it has not already, with the declining chloroquine use.

Chloroquine monotherapy continues to demonstrate modest therapeutic utility in west Africa. A recent observational study in Benin examined the effect of self-administered chloroquine chemoprophylaxis among pregnant women (N = 1090), comparing self-reported dosing over pregnancy with birth weights. An estimated 49.9% of women reported taking weekly chloroquine in the first trimester, increasing to 92% of women in the second trimester and 97.5% in the final trimester. Random testing of urine samples at delivery established a point-prevalence for chloroquine use. In total, an estimated 99% of women had ingested chloroquine (N = 166); of these, 72% had levels consistent with consuming 300 mg in the previous seven days. Subjects with self-reported chemoprophylactic use for seven or more months were four times more likely to give birth to child of normal birth weight (> 2500 grams) than women who used chemoprophylaxis for less than four months (adjusted OR = 3.96; 95% CI = 1.9 to 8.28; p =< 0.001) [63].

Parasitological evidence of chloroquine efficacy was reported, as well, in a recent four-arm clinical trial conducted in Ghana among pregnant women with asexual *P falciparum *stage parasitaemia. Women randomized to a chloroquine treatment group (N = 225) received 600 mg for 2 days and 300 mg on the third day. The uncorrected day-28 treatment failure rate was 30% (62 of 208). Polymerase chain reaction (PCR) analysis confirmed that 14% (30 of 208) were treatment failures while 6% (11 of 208) were re-infections. PCR was unable to distinguish cases of recrudescence from new infection in the remaining 10% (21 of 208) [64].

### Potential for azithromycin and chloroquine when used in combination for the prevention of malaria

#### Evidence for synergy of the combination in vitro

An additive effect between azithromycin and chloroquine has been shown in sensitivity testing conducted over a 48-hour period. When incubation is extended to 68 hours, drug synergy has been seen against chloroquine-resistant isolates; the combination remains additive, however, against chloroquine-sensitive parasites [65]. Sidhu *et al *in contrast, observed an additive effect at 96 hours of incubation against chloroquine-resistant isolates [23].

Azithromycin and chloroquine do not exhibit any clinically relevant pharmacokinetic interactions [66], although chloroquine resistance is reversible with calcium channel blockers, such as verapamil and desipramine, that inhibit p-glycoprotein-mediated efflux [67,68]. Azithromycin is a p-glycoprotein substrate [69] which may suggest the presence of a metabolic mechanism for synergy. Whether additive or synergistic, complete parasitological clearance using the combination would not be expected in less than 48 hours, the equivalent of two schizogenesis cycles. Thus, a conservative approach to *in vivo *dosing may require a three-day regimen to realize the full benefits of the azithromycin-chloroquine combination against *P. falciparum *while minimizing the opportunity for survival of wild-type progeny.

#### Evidence for synergy of the combination in vivo

A two-stage trial in India demonstrated *in vivo *synergy between azithromycin and chloroquine against *P. falciparum *infection. The first stage, which was double-blinded, included 32 semi-immune subjects treated for uncomplicated *P. falciparum *malaria with either azithromycin (1,000 mg) plus chloroquine placebo on days 0, 1 and 2, or chloroquine (600 mg) on days 0, 1 and 300 mg on day 2 plus azithromycin placebo on days 0, 1 and 2. The second stage of the trial was open label and included 64 semi-immune subjects who received the azithromycin-chloroquine combination therapy in doses equal to stage one. Treatment response rates at day 28 showed *in vivo *synergy: 33% of those who received azithromycin monotherapy remained free of fever by day 28 compared to 27% in the chloroquine-treatment group. In contrast, 97% of patients who received drugs co-administered had resolved fever and parasitaemia by day 7 with no observed recrudescence by day 28. Parasitological responses by treatment group mirrored the synergy of clinical observations. Azithromycin monotherapy eradicated parasites in 19% of subjects (3 of 16) by day 3, increasing to 63% of subjects (10 of 16) by day 7, and dropping to 36% at day 28. As would be expected, chloroquine monotherapy was faster-acting than azithromycin alone, but it also demonstrated an increase in failures by day 28. Specifically, 56% of subjects (9 of 16) were free of parasites at day 3, followed by 88% (14 of 16) at day 7 and, finally, 27% (4 of 15) by day 28. For subjects receiving azithromycin-chloroquine combination treatment, however, 97% parasitological eradication was achieved by day 3 and sustained through day 7 and day 28 [70].

### Treatment trials with the azithromycin-chloroquine combination in Africa

A double-blinded multi-centre trial was held in Burkina Faso, Ghana Mali, Kenya, Uganda, and Zambia to compare the therapeutic efficacy and tolerability of azithromycin-chloroquine with that of mefloquine [71]; a second, open-label, confirmatory trial was conducted in the same countries, with the addition of Senegal [72]. Together, these studies established an efficacious treatment course for azithromycin-chloroquine against uncomplicated *P. falciparum *infection: a fixed daily dose of 1,000 mg of azithromycin and 600 mg of chloroquine taken for three days. This treatment regimen represents a slightly higher dose of chloroquine than has been commonly administered. Most often, 25 mg of chloroquine is provided per kg of body weight over a three-day period: 10 mg per kg on days 1 and 2 with 5 mg per kg on day 3. The azithromycin-chloroquine fixed dose contains a total of 1,800 mg of chloroquine, an amount that would typically be given to a person weighing 72 kg. The average weight of pregnant women in the Ghana efficacy study [64] was 55.9 kilograms. Thus, 600 mg per day over three days is 22.4% more chloroquine than is in the typical treatment course.

Mefloquine was an appropriate comparator in these sub-Saharan studies because it has not been used regularly in the region and parasite sensitivity is likely quite high. Mefloquine is also a potential candidate to replace SP in IPTp. Preliminary results suggest that azithromycin-chloroquine is non-inferior to mefloquine with *P. falciparum *clearance rates comparable to those observed in India. The azithromycin-chloroquine combination warrants further investigation and may also offer particular advantages in settings where mixed infections of *P. falciparum *and *P. vivax *predominate.

### The azithromycin-chloroquine combination for IPTp

#### Dosing

If azithromycin and chloroquine are used together in IPTp, then an initial priority should be to identify the most suitable dose of the combination. It seems likely that a three-day treatment regimen will be needed to ensure complete parasitological clearance while minimizing selection for resistant genotypes. Thus, as an initial investigation, it would be appropriate to give women two or three courses of IPTp during the antenatal period in a regimen of 1,000 mg of azithromycin plus 600 mg chloroquine base, once daily for three days. Because rates of drug absorption, distribution and excretion are commonly altered during pregnancy, pharmacokinetic investigations should be conducted as part of, or in parallel to, a clinical trial.

#### Acceptance and adherence

Acceptance of and adherence to a three-day regimen of azithromycin-chloroquine would be needed. In most operational settings, the first dose of azithromycin-chloroquine can be administered as directly observed therapy during antenatal visits, but doses on the following two days would require self-administration. Improvements in adherence to multi-day regimens have been shown for discrete treatment periods using pre-packaged sachets labeled with pictogram instructions that are explained during initial consultations [73-76]. Even so, achieving high rates of adherence to a three-day azithromycin-chloroquine regimen – administered two or three times during pregnancy – would likely be a challenge. Public education campaigns in recent years have discouraged chloroquine use. Thus, community acceptance, even in a new combination, would require innovative packaging and marketing. Adherence could be improved if a three-dose azithromycin-chloroquine fixed-dose formulation is designed specifically for IPTp. In countries currently implementing Home-based Management of Malaria and/or the community component of the Integrated Management of Childhood Illness, adherence to IPTp could be improved further with community health workers making house visits on days following antenatal consultations to verify self-treatment while taking the opportunity to develop or review an individualized perinatal plan in the home.

Azithromycin plus SP is another option for IPTp which would not present the disadvantage chloroquine-associated pruritus. However, SP may have surpassed a resistance threshold which would make it an ineffective partner drug. Alternatively, azithromycin could be combined with piperaquine to improve adherence; piperaquine is at least as effective as, and better tolerated than, chloroquine against *P. falciparum *and *P. vivax *[77]. Pyronaridine is another potential partner drug, shown to have additive properties with azithromycin [78]. Mefloquine, despite some important issues of tolerability, has the advantage that it can be administered as a single, observed dose.

#### Optimal timing

SP is contraindicated prior to quickening due to its teratogenic risk and, again, one month prior to delivery because of possible drug-induced kernicterus. Thus, providing IPTp with SP requires estimating gestational age and delivery date with some accuracy. In contrast, there are no known contraindications for azithromycin-chloroquine at any gestational age. This is important because current IPTp guidelines are currently based on operational convenience rather than the natural course of MIP [79]. Earlier IPTp administration may be important as maternal parasite densities peak between nine and 16 weeks [1,3], tapering until term. IPTp administration in the last month of pregnancy may be of considerable value, too, increasing foetal weight gain during final stages of accelerated growth *in utero *[79].

Mefloquine may also be suitable for administration earlier and later in pregnancy than SP. Based, in part, on post-marketing surveys and retrospective studies which include 1271 first-trimester pregnancies, the US Centers for Disease Control and the UK Health Protection Agency recommend mefloquine chemoprophylaxis for pregnant women in any trimester when travelling to areas of high malaria transmission. Overall experience does not suggest that mefloquine is teratogenic [16,80]. Administration in the first trimester, however, may warrant caution in light of two retrospective studies that found associations between mefloquine exposure and spontaneous abortion [81], and stillbirth [80].

ACTs are associated with embryotoxicity over a narrow dose range in animal models of early pregnancy with some additional evidence of lethality in second and third trimesters [82]. For this reason, the WHO recommends ACTs for curative purposes only during the second- and third-trimesters if other treatments have been considered unsuitable. First-trimester administration is contraindicated unless treatment is considered life-saving for the mother [83].

### Potential additional benefits of azithromycin-chloroquine when used for IPTp

Use of the azithromycin-chloroquine combination in IPTp may offer several additional public health benefits over other possible replacements for SP.

#### Reduction of sexually transmitted infections

Sexually transmitted infections (STI) adversely affect pregnancy and contribute to pre-term delivery, LBW, intrauterine growth-retardation, spontaneous abortion, stillbirth, newborn morbidity and mortality [84]. Maternal health is also jeopardized by STIs with potential complications including pelvic inflammatory disease, ectopic pregnancy and infertility [85]. Prevalence estimates of symptomatic STIs at antenatal clinics in sub-Saharan Africa range between 2.5 and 17% for syphilis [86-91], 1.7 and 7% for *Neisseria gonorrhoea *[86-88,91-96], 5.3 to 20.8% for *C. trachomatis *[86-88,92-95,97] and 7.3% to 62% for chancroid [98].

In resource-limited settings, testing women for STIs during antenatal consultations and providing appropriate care has been a public health challenge for decades. To assist countries, the WHO has developed syndromic-based algorithms for the detection of STIs. In high-transmission areas, the method is reliable for men, but much less so for women. Syndromic diagnosis of *N. gonorrhoea *and *C. trachomatis *among women has a sensitivity of 30 to 80% and a specificity of 40 to 80%; rarely does the sum of the two exceed 120% [99-102]. An additional shortcoming is that asymptomatic infection, a substantial portion of disease burden, remains undetected and, thus, untreated.

In South Africa's largest district, Hlabisa, 24.9% of females between 15 and 49 years of age are estimated to have at least one STI on any given day – *Treponema pallidum, N. gonorrhoea, C. trachomatis*, or *Trichomonas vaginalis*. While this prevalence may be higher than in some other parts of sub-Saharan Africa, of particular concern is that 48% of these infections are asymptomatic. In addition, just 2% of women with symptomatic STIs ever seek treatment in Hlabisa, and when they do, only 65% of them receive adequate care. Pregnant women are less likely to have asymptomatic STIs compared to non-pregnant women (17% vs. 59%), but the age-specific prevalence of infection is often twice as high for pregnant women [103]. This level of disease prevalence, symptomatic and asymptomatic, suggests a role for mass treatment during pregnancy.

The presumptive treatment of STIs in pregnancy improved maternal health and birth outcomes in a randomized clinical trial involving 4,033 pregnancies in Uganda [88]. Vaginal infections were significantly lower in women who received a one-time dose of azithromycin (1,000 mg), metronidazole (2,000 mg) and cefixime (400 mg) compared to women who received iron/folate and low-dose multivitamins. In the treatment group, the relative risk (RR) of *T. vaginalis *was 0.28 (95% CI, 0.18–0.49), the RR of bacterial vaginosis was 0.78 (95% CI, 0.69–0.87), and the RR of infant ophthalmia was 0.37 (95% CI, 0.20–0.70). The incidence of LBW was substantially reduced in the intervention group (RR, 0.68; 95% CI, 0.53–0.86) as was early neonatal mortality (25.4 per 1,000 live births), when compared to the control group (29.1 per 1,000 live births).

While difficult to attribute specific beneficial outcomes to each compound, azithromycin probably had a considerable effect. An oral dose of 1,000 mg of azithromycin clears more than 90% of cervical infections due to *N. gonorrhoea *and *C. trachomatis *[85]. The same dose will cure and provide chemoprophylaxis against chancroid and syphilis. Studies in Uganda [104] and Tanzania [105] have shown that azithromycin, 1,000 mg and 2,000 mg respectively, is equally effective as benzathine penicillin G in treating syphilis among non-pregnant adults. If untreated in pregnancy, one-third of women will develop congenital syphilis, carrying major risk for the foetus. One study in Tanzania found unscreened congenital syphilis associated with 51% of stillbirths, 24% of pre-term live births and 17% of adverse pregnancy events [90]. Another trial in Zambia implicated maternal syphilis in 42% of spontaneous abortions [106]. Despite the high cure rates observed in the clinical trials of Uganda and Tanzania, 1,000 mg and 2,000 mg of azithromycin given to five pregnant women with syphilis in China did not prevent trans-placental infection [107]. Thus, IPTp with azithromycin-chloroquine should not be viewed as a replacement for benzathine penicillin G in the prevention of congenital disease. However, azithromycin-chloroquine administered in IPTp may improve outcomes for the majority women whose syphilis infections, both symptomatic and asymptomatic, that are currently undiagnosed and untreated.

The extent to which the control of STIs can prevent the spread of HIV remains unknown. Observational studies associate treatment of ulcerative STIs with reductions in HIV transmission, particularly among men [108], and yet a systematic review of eight clinical trials failed to find the same relationship in seven of them [109]. It is possible that two or three IPTp treatments with azithromycin-chloroquine could offer women some protection against HIV infection in pregnancy, but the observable difference may be undetectable due to sample size limitations in most clinical trials, especially in areas with low HIV prevalence rates.

Chloroquine may offer its own protection against HIV transmission. Cord blood containing high levels of chloroquine has been associated with a reduced risk of mother-to-child transmission (MTCT) of HIV [110]. In addition, viral shedding in breast milk has been lowered among HIV-positive women who received three days of 600 mg chloroquine as an anti-malarial chemoprophylactic [111]. It is unknown whether this reduction in viral load is sufficient to prevent HIV transmission among mothers who choose to breastfeed.

#### Prevention of pneumococcal infection in pregnancy

Pneumonia is not a common focus of maternal health packages in most resource-limited settings because the incidence in pregnancy is not appreciably higher than in non-pregnant women [112]. Disease progression, however, is substantially more virulent during gestation [113-115]. There are old data from Ibadan, Nigeria, that suggest the incidence of pneumococcal meningitis may increase during pregnancy and puerperal period [116]. Facility records between 1958 and 1962 revealed that 86% (26 of 31) of women with pneumococcal meningitis were pregnant (15) or had recently delivered (11). By comparison, disproportionately fewer pregnant or early postpartum women, 22% (7 of 32) in total, were diagnosed with other types of meningitis. It is uncertain whether this enhanced risk in pregnancy occurs in other parts of Africa. If so, then it is conceivable that IPTp with azithromycin might provide some protection against this uncommon but very serious infection.

### Cost

The cost of 1,800 mg of chloroquine (600 mg per day for three days) ranges between US $0.10 and $0.20, while 3,000 mg of azithromycin (1,000 mg per day for three days) is approximately US $6.80 [117]. Thus, an azithromycin-chloroquine IPTp regimen administered two or three times would cost between US $14.00 and $21.00 per pregnancy. This is prohibitively expensive for national malaria control programmes in most endemic countries in Africa. Thus, external funding would be required for widespread implementation of the combination in IPTp. This could take many forms: a direct donation from the pharmaceutical industry, and/or a financing mechanism modelled after the Global ACT Subsidy or the International Financing Facility for Immunization.

### Azithromycin and selection for resistance

There are concerns that use of azithromycin-chloroquine for IPT could encourage the emergence and spread of resistance to a variety of organisms. Pathogens that need to be considered include malaria parasites, organisms causing STIs, and the pneumococcus.

#### Malaria parasites

Apart from clinical trials, azithromycin has never been used operationally for treatment or prevention of malaria. The susceptibility of azithromycin-chloroquine for selecting parasites resistant to azithromycin is, therefore, unknown. The declining use of chloroquine throughout sub-Saharan Africa is likely to lead to reversal of resistance as witnessed in Malawi where parasite sensitivity returned five years after suspending its use. It is possible the re-introduction of chloroquine with azithromycin as a partner drug may prevent re-selection of parasites carrying the *pfcrt *resistance mutation. However, rigorous surveillance would be needed to verify this assumption.

#### Organisms causing STIs

Azithromycin sensitivity patterns in high-income countries do not suggest that its use in IPTp would rapidly induce resistance in *N. gonorrhoea *or *C. trachomatis*. Sensitivity of the gonococcus remains relatively high, even in the presence of growing penicillin resistance. In the United States, minimum inhibitory concentrations (MICs) of azithromycin exposed to gonococcal isolates have increased modestly since 1992 when tracking began. In 2006, 0.2% (14 of 6,089) of isolates provided through a national network were resistant to azithromycin with a MIC > 2.0 μg/ml, representing a slight decrease from 0.6% (35 of 6,199) of isolates in 2005 [118]. In the case of *C. trachomatis*, the most recent meta-analysis of 12 trials involving 1,543 patients estimates cure rates to be 97% with a single 1,000 mg dose of azithromycin [119].

There is greater concern regarding syphilis. Azithromycin has been used, for example, since 1999 in San Francisco (USA) for chemoprophylactic (1,000 mg) and curative (2,000 mg) purposes against syphilis. By 2004, a mutation associated with *T. pallidum *resistance to macrolides at the A2058 position in the 23S rRNA gene was found in 56% blood samples from the main metropolitan sexual health centre. All isolates were from men who have sex with men, 31% of which were from HIV-infected men [120]. The rapid decline in sensitivity is likely attributable, in large part, to underlying erythromycin resistance which has been in general use for over 50 years. Erythromycin-resistant *T. pallidum *isolates with mutation at the A2058 position confer resistance to macrolide antibiotics and are associated with treatment failures [121,122]. Indeed, a risk factor for being infected with azithromycin-resistant syphilis is having used azithromycin or other macrolides in the recent past [123,124].

The potential for rapid induction of *T. pallidum *resistance in Africa is difficult to estimate since molecular analyses of the A2058 region have not been included in most syphilis studies of the region. In Madagascar, the mutation was not found in analysis of 103 *T. pallidum *isolates and no azithromycin treatment failures have been reported in country [123,125]. Because neither macrolide has been deployed on any scale in focus countries of IPTp, azithromycin may be less vulnerable to rapid loss of sensitivity as has been witnessed in high-income countries.

#### The pneumococcus

Pneumococcal resistance to macrolides occurs by two primary mechanisms, each with distinct genetic markers: ribosomal methylation (*ermB *or *ermA *genes) and efflux pump mutation (*mefA *or *mefE *genes). Based on experience in mass treatment of trachoma with azithromycin, there is concern that use of the azithromycin-chloroquine combination for IPTp might increase the prevalence of azithromycin- and erythromycin-resistant pneumococci. Trachoma eradication campaigns using azithromycin among vulnerable groups of children in Australia and Nepal found that one-time treatment may select macrolide-resistant pneumococcal strains in the nasopharynx [126,127] and conjunctiva [128]. Selection, however, was transient. In Australia, 98.7% (78 of 79) of nasopharyngeal pneumococcal isolates collected at baseline were sensitive to azithromycin, decreasing to 84.2% (32 of 38) between two to three weeks, and then 73% (27 of 37) at two months. By six months, 94.9% of isolates were sensitive to azithromycin [126]. One-year after a trachoma campaign in Nepal, 86% (50 of 57) of randomly collected isolates were positive for *S. pneumoniae*, and 100% (50 of 50) were azithromycin sensitive [129]. Selection of azithromycin-resistant strains, however, has not always followed trachoma treatment campaigns. Very high macrolide sensitivity of nasopharyngeal pneumococci was observed in Tanzania when samples were obtained at three weeks, two months and six months post-treatment. Of 4,782 pneumococcal swabs tested, only one demonstrated pneumococcal resistance to azithromycin. Curiously, the resistant sample was collected at six months, not earlier, as might be expected with a treatment-induced mutation [130].

### Ways to reduce opportunity for resistance

A number of steps could be taken to reduce the chances that azithromycin use in IPT might enhance macrolide resistance in bacteria responsible for several major infections. Considerations include the following:

#### Setting dose and duration above resistance breakpoint

A counter-selective dose – the minimum dosage necessary to prevent the emergence of drug resistance – can be used instead of the conventional minimum required dose to achieve adequate clinical and parasitological cure. Such a dose, set just above the resistance breakpoint of target micro-organisms, would suppress most pathogens during initial drug exposure and sustain concentrations sufficient to inhibit mutant progeny that might survive and select for resistance. Breakpoints and a counter-selective dose for azithromycin-chloroquine can be modelled using pharmacokinetic and pharmacodynamic parameters in IPTp target countries. While there will always be the potential for inducing resistance, a multi-national, multi-centre surveillance study over 10 years has shown that treatment of respiratory tract infections to the point of bacterial eradication minimizes potential for selecting and maintaining resistant strains [131].

#### Limiting availability

If azithromycin-chloroquine is limited to IPT, made available only through health facilities, and not simultaneously used for treatment purposes, then drug pressure can be kept to a minimum.

#### Monitoring sensitivity of pneumococci

Coordinated resistance surveillance of pneumococci should become a regional objective if countries choose to adopt azithromycin-chloroquine for IPT. Regional networks already exist for monitoring malaria and pneumococcal resistance, and could collaborate on this objective. Monitoring, however, can be a source of controversy as the relevance of *in vitro *macrolide sensitivity to clinical outcomes is not well established. There has not been a concomitant rise in *S. pneumoniae *case-mortality rates as increasing macrolide resistance has been observed *in vitro *[132]. A similar paradox has been seen with penicillin-resistant *S. pneumoniae *[133,134]. Multiple reasons may contribute to these discordant trends. In the case of newer macrolides, including azithromycin, drug concentrations are able to reach higher levels in the intracellular tissue and in the epithelial lining fluid of the lung than concentrations measured in blood [135,136]. Azithromycin, therefore, may have superior pharmacokinetics *in vivo *to inhibit *S. pneumoniae *infection, safeguarding favourable treatment outcomes in the face of increasing macrolide resistance as measured *in vitro*. However, macrolide efflux pump mutations have been identified in *S. pneumoniae *isolates with erythromycin MICs of at least 8 μg/mL – concentrations of azithromycin that have been associated with clinical failures [137]. The *in vitro-in vivo *paradox may be better understood with improved surveillance that involves analysis of *in vitro *MICs and *in vivo *treatment outcomes that include morbidity markers – not just morbidity rates – which may be more sensitive in detecting the effect of *in vitro *changes in macrolide resistance on clinical outcomes [134].

### Key scientific and programmatic questions

Evidence to date suggests that azithromycin-chloroquine is a potential alternative for SP for IPTp and its evaluation in clinical trials is warranted. Several scientific and programmatic questions need to be addressed, however, so that policymakers and programme managers are able to consider the merits of azithromycin-chloroquine in IPTp. Key questions include:

1) Is azithromycin-chloroquine superior to SP and other candidate replacements for IPTp in reducing LBW, maternal anaemia and parasite clearance?

2) How much of the IPTp effect on birth weight, using azithromycin-chloroquine, may be due to a reduction in STIs, and what might be the savings, human and financial, due to reduced STIs in pregnancy that could result?

3) Might administration of azithromycin-chloroquine prior to quickening and within the last month of gestation – periods contraindicated with SP – have additional effect on LBW, maternal anaemia and parasite clearance?

4) Would the use of azithromycin-chloroquine for IPTp result in more than transient changes in the sensitivity of pneumococci to macrolides and penicillin?

5) How might countries monitor the effect of azithromycin-chloroquine on the sensitivity of pneumococci to macrolides?

6) What is the counter-selective dose for azithromycin-chloroquine against malaria parasites, organisms causing STIs, and pneumococci?

7) How would pregnant women respond to receiving azithromycin-chloroquine and adhere to a regimen that requires partial self-administration, particularly as information campaigns have discouraged chloroquine use in recent years?

8) Might IPTp with azithromycin-chloroquine reduce maternal acquisition of HIV during pregnancy, *in utero *MTCT or post-partum transmission among sero-positive women who choose to breastfeed?

## Conclusion

Azithromycin-chloroquine is a potential alternative to SP for use in IPTp. The combination has demonstrated synergism *in vivo *against *P. falciparum *in India. Preliminary results of studies in non-pregnant adults in sub-Saharan Africa have shown that azithromycin-chloroquine is not inferior to mefloquine, a compound currently under consideration for IPTp. The azithromycin-chloroquine combination may be safely administered at any time in pregnancy. The secondary benefits of the combination, clearing of symptomatic and asymptomatic STIs, may be as important to maternal, foetal and neonatal health as the clearance and prevention of malaria. Innovative pricing mechanisms would be required to introduce azithromycin-chloroquine for IPTp since the drug cost per pregnancy would otherwise be US $14.00 to $21.00 for two or three courses. Close monitoring of antibiotic resistance markers would need to be an essential part of any IPTp programme using azithromycin-chloroquine.

## Competing interests

The authors declare that they have no competing interests.

## Authors' contributions

MC structured the review and wrote the paper. RP reviewed the early manuscript and contributed to the writing of the paper. BG reviewed the early manuscript and contributed to the writing of the paper. DC refined the structure of the review and contributed to the writing of the paper. All authors read and approved the final version.
